# Crystal Growth of 3D Poly(*ε*‐caprolactone) Based Bone Scaffolds and Its Effects on the Physical Properties and Cellular Interactions

**DOI:** 10.1002/advs.202203183

**Published:** 2022-11-17

**Authors:** Boyang Huang, Yaxin Wang, Cian Vyas, Paulo Bartolo

**Affiliations:** ^1^ Singapore Centre for 3D Printing School of Mechanical and Aerospace Engineering Nanyang Technological University Singapore 639798 Singapore; ^2^ School of Mechanical Aerospace and Civil Engineering University of Manchester Manchester M13 9PL UK

**Keywords:** mechanotransduction, melt printing, solvent printing, strain‐induced crystallization, temperature‐induced crystallization

## Abstract

Extrusion additive manufacturing is widely used to fabricate polymer‐based 3D bone scaffolds. However, the insight views of crystal growths, scaffold features and eventually cell‐scaffold interactions are still unknown. In this work, melt and solvent extrusion additive manufacturing techniques are used to produce scaffolds considering highly analogous printing conditions. Results show that the scaffolds produced by these two techniques present distinct physiochemical properties, with melt‐printed scaffolds showing stronger mechanical properties and solvent‐printed scaffolds showing rougher surface, higher degradation rate, and faster stress relaxation. These differences are attributed to the two different crystal growth kinetics, temperature‐induced crystallization (TIC) and strain‐induced crystallization (SIC), forming large/integrated spherulite‐like and a small/fragmented lamella‐like crystal regions respectively. The stiffer substrate of melt‐printed scaffolds contributes to higher ratio of nuclear Yes‐associated protein (YAP) allocation, favoring cell proliferation and differentiation. Faster relaxation and degradation of solvent‐printed scaffolds result in dynamic surface, contributing to an early‐stage faster osteogenesis differentiation.

## Graphical abstract

Melt and solvent extrusion‐based additive manufacturing technologies are used to fabricate the 3D scaffold for bone tissue engineering. In the printing process, the polymer crystal growth is strongly associated with temperature‐induced crystallization and strain‐induced crystallization, leading to distinct physiochemical properties. These properties have a strong impact on the cell–substrate interactions by mechanotransduction pathways.

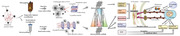

## 1 Introduction

Tissue engineering combining additive manufacturing (AM), biomaterials, biomolecules, and cells has emerged as a promising strategy in the field of bone repair and extrusion‐based 3D printing is the most commonly used approach to fabricate customized and porous bone constructs using natural and synthetic polymer materials.^[^ ^1^, ^2^, ^3^, ^4^ ^]^ Normally, extrusion‐based additive manufacturing techniques are classified by the fluid‐dispersing configuration, as pneumatic‐, mechanical‐ (piston or screw‐driven) or solenoid‐based system.^[^ ^5^ ^]^ The selection of the appropriate extrusion‐based AM for printing is also strongly associated with the material compositions where the consideration of printing temperature is consequently inevitable. Melt extrusion AM has been extensively used in tissue engineering to process synthetic thermoplastic polymers and polymer‐based composites at relatively high temperatures, which poses some limitations regarding the use of temperature‐sensitive biomolecules.^[^ ^6^ ^]^ Solution/solvent‐based extrusion 3D printing is a more recent alternative allowing to print inks, based on a range of natural and synthetic polymers and suitable solvents, at low/room temperatures.^[^ ^7^, ^8^, ^9^, ^10^, ^11^ ^]^ This technique allows the incorporation of temperature‐sensitive biomolecules and a high loading of bioactive particles.^[^ ^9^, ^12^, ^13^ ^]^ However, a limited number of articles reported a comparative study between melt and solvent printing.^[^ ^10^, ^14^, ^15^, ^16^ ^]^ Moreover, these studies mostly focused on the optimization of printing parameters or on the physical characteristics of printed scaffolds. The mechanism of inducing distinct physical properties on these two different types of scaffolds remains unknown.

Cells perceive the microenvironment through biophysical and biomechanical factors such as substrate surface topography, stiffness, and degradability.^[^ ^17^, ^18^ ^]^ The mechanotransduction pathway allows cells to directly respond to mechanical stimuli and regulate cell behaviors such as cell proliferation and differentiation. The mechanotransduction regulating mesenchymal stem cells (MSCs) cell fate has been extensively studied in 2D environments.^[^ ^19^, ^20^, ^21^ ^]^ However, cells behave completely different in a 2D or 3D environment. Moreover, studies addressing 3D mechanobiology are mostly conducted within hydrogels with a substrate stiffness below 500 kPa.^[^ ^22^, ^23^, ^24^, ^25^ ^]^ The mechanotransduction pathways using additive manufactured 3D scaffolds made with high stiffness polymers are still largely unexplored.

Polycaprolactone (PCL) is a synthetic semicrystalline thermoplastic polyester approved for use in biomedical applications by the Food and Drug Administration (FDA). It has been widely used in bone tissue engineering applications due to its flexibility in processing (low melting temperature and good solubility), controllable degradation (up to four years), and tunable mechanical properties.^[^ ^4^, ^26^ ^]^ Moreover, PCL has excellent blend compatibility allowing development of a variety of bioactive composites and scaffolds to mimic bone and promote regeneration.^[^ ^27^ ^]^ For example, tricalcium phosphate (TCP) is an osteoconductive bioceramic material used in PCL‐based scaffolds, although considered to be less osteoinductive than other bioceramics such as hydroxyapatite (HA). The biodegradation and bioresorbability still make it attractive for bone tissue engineering.^[^ ^28^ ^]^ Moreover, our previous study demonstrated the better miscibility of TCP in a PCL matrix compared to HA.^[^ ^29^ ^]^

Bone is a highly porous, mineralized, and viscoelastic tissue exerting fundamental roles in the human body such as support, protection of other tissues and performing mineral homeostasis.^[^ ^30^ ^]^ The use of biopolymers, bioceramics, and appropriate AM technologies to fabricate 3D porous scaffolds is highly relevant to mimic these properties. This work used melt and solvent extrusion AM to fabricate PCL based scaffolds for bone tissue engineering. TCP particles were blended with the polymer matrix providing biomechanical and biochemicues. The characteristics of the two different types of printed scaffolds were deeply investigated, as well as the influence of the scaffold's physical features on the mechanotransduction. Moreover, this study provides an insight into the process‐physiochemical‐biological relationship when using biomaterial inks for extrusion‐based AM applications in bone tissue engineering and a fundamental study of mechanobiology in a 3D environment.

## 2 Results

### 2.1 Physiochemical Properties of Melt and Solvent Extruded 3D Printed Scaffolds

**Figure 1:**
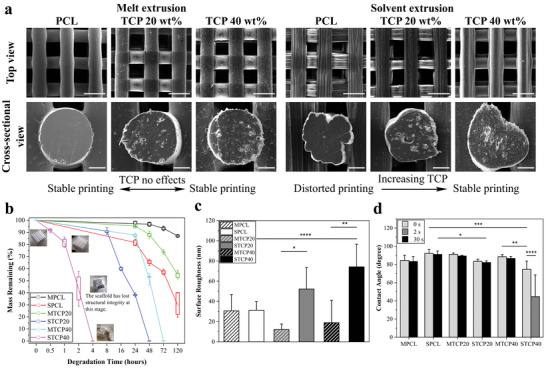
Physical properties of printed scaffolds. a) SEM images showing top and cross‐sectional views of melt and solvent printed scaffolds (scale bar: top view, 500 µm; cross‐sectional view, 100 µm). b) Accelerated degradation profiles and mass remaining up to 120 h. The inset images show loss of structural integrity of the scaffold after 50% mass loss (*n* = 5). c) Surface mean roughness *R*_a_ of the scaffolds, considering a 50 µm × 50 µm surface area (*n* ≥ 5). d) Water contact angle measurements at 0 and 30 s, and additional measurement at 2 s for STCP40 (*n* = 3). Data were presented as mean ± standard deviation (SD) for b, c and d. Statistical analysis was performed using one‐way analysis of variance (ANOVA) for c and d. Differences were considered significant at *P < 0.05, **P < 0.01, ***P < 0.001 and ****P < 0.0001.

3D porous PCL‐based scaffolds with well‐defined and uniform structures were successfully fabricated using both melt and solvent extrusion AM (**Figure** **1**a). Melt and solvent printed PCL only scaffolds are referred to as MPCL and SPCL, respectively. The scaffolds share a similar morphology (filament diameter, pore size, and layer thickness) and closely match the designed parameters. Moreover, all scaffolds present a similar porosity of ≈53% (Table S1, Supporting Information). However, these two printing approaches result in printed filaments with significantly different appearances. For all material compositions, the melt‐ printed filaments exhibit uniform circular cross‐sections and smooth surfaces, indicating a stable printing process. Contrary, the solvent‐extruded filaments present a flake‐like grooved surface morphology and irregular cross‐sections. However, results observed from solvent‐printed PCL/TCP 80/20 wt% (STCP20) and 60/40 wt% (STCP40) scaffolds seem to indicate that by increasing the TCP content 3D printed filaments present a more circular cross‐section and smoother surface. It is also possible to observe that the TCP particle distribution is similar in both melt and solvent extruded filaments at 20 wt% loading. However, at 40 wt% a noticeable difference is observed with the STCP40 scaffolds presenting a more homogenous distribution and smaller particle aggregation (Figure S1, Supporting Information). It is known that the printability of the scaffolds is strongly dependent on the rheological behavior of the composite material.^[^ ^29^ ^]^ Experimental results show that the solvent blending method allows high loading conditions, up to 60 wt% in the PCL matrix, while the melt blended composite containing 50 wt% TCP presented poor printability due to the “liquid‐like” to “solid‐like” phase transition (Figure S2, Supporting Information). Therefore, the TCP concentration was limited to 40 wt% to enable both melt and solvent extrusion to operate in the printable region.

The degradation profile of the scaffolds was obtained using an accelerated chemical degradation process (Figure 1b). Overall, the solvent‐printed scaffolds have a faster degradation rate than the melt‐printed scaffolds. MPCL scaffolds present the best structure stability, with 87% of the mass remaining after 120 h. Moreover, the degradation kinetics increase by increasing the TCP concentration. Melt‐printed PCL/TCP 60/40 wt% (MTCP40) and all solvent‐printed scaffolds lost their structural integrity within 5 d. In particular, STCP40 presents the fastest degradation rate with structural integrity lost after 2 h of immersion in the degradation solution. Although accelerated degradation studies are used to elucidate potential degradation profiles of a material in both physiological in vitro and in vivo environments, the results must be analyzed with caution. The degradation profile of PCL in alkali accelerated degradation and physiological degradation are both through hydrolytic random scission but proceed via surface and bulk pathways, respectively.^[^ ^31^, ^32^ ^]^ This results in a distinct degradation profile that is not comparable to physiological in vitro and in vivo degradation profiles.^[^ ^32^, ^33^ ^]^ Subsequently, long‐term physiological in vitro or animal studies should be used to understand biologically relevant degradation profiles, which is out of the scope of this study. However, this study uses accelerated degradation as a tool to understand the relationship between physiochemical properties (e.g., crystal microstructure), as a function of printing process, and degradation.

The surface topography of the scaffolds was measured through atomic force microscopy (AFM) (Figure 1c and Figure S3, Supporting Information), and the results show no statistical difference between MPCL and SPCL scaffolds. However, the presence of TCP significantly alters the surface roughness of scaffolds. In the case of melt printed scaffolds the increase in the TCP content decreases the surface roughness, with the melt printed PCL/TCP 80/20 wt% (MTCP20) presenting the lowest *R*_a_ (≈10 nm). However, in the case of solvent printed scaffolds results show that the surface roughness increases by increasing the TCP content, with STCP40 scaffolds exhibiting the highest *R*_a_ (≈70 nm) and significant micropores at the filament surfaces (Figure S3g, Supporting Information). This might be attributed to the formation of micropores/microchannels on the scaffold surface as shown in Figure 1a and Figures S1 and S3 (Supporting Information). From these figures, it can be observed that the micropores/micro‐channels are formed by the collision of adjacent microcrystal growth on MPCL surface, but this phenomenon is mitigated with the increase of TCP concentration. In terms of solvent‐printed scaffolds, more pores/channels are observed with the increase of TCP, especially in STCP40, increasing surface roughness.

The wettability of the MPCL and SPCL scaffolds are ≈83° and 91°, respectively (Figure 1d and Figure S4, Supporting Information), indicating that the MPCL scaffolds are slightly hydrophilic and the SPCL slightly hydrophobic. However, no significant differences were observed between sample types. The water contact angle (WCA) of the melt‐printed scaffolds slightly increases by increasing the TCP loading, with the MTCP20 scaffolds presenting the highest WCA values. Inversely, the solvent‐ printed scaffolds present a decreasing trend of WCA with increasing the TCP loading. As observed, the STCP40 scaffolds display superhydrophilicity with a WCA of ≈75° at 0 s and the water droplet being fully absorbed at 30 s. In this case, an additional droplet measurement was conducted at 2 s with a WCA of ≈45°.

**Figure 2:**
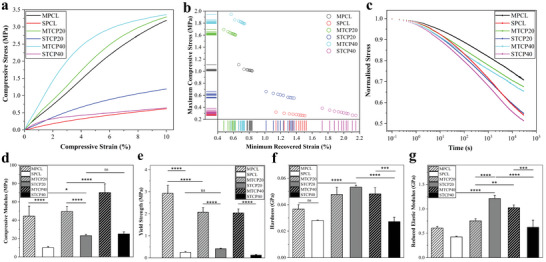
Mechanical properties of printed scaffolds. a) Representative compressive stress–strain curves for bulk mechanical properties of the scaffolds, b) maximum compressive stress at minimum recovered strain of cyclic compression for all scaffolds, and c) stress relaxation tests of the scaffolds at 10% compressive strain. Stress is normalized to the maximum stress. d) Compressive modulus (*n* = 6) and e) yield strength of the scaffolds (*n* = 6). f) Hardness (*n* = 3) and g) reduced elastic modulus of the scaffolds determined by nanoindentation (*n* = 3). Data were presented as mean ± SD for d to g. Statistical analysis was performed using one‐way analysis of variance (ANOVA) for d to g. Differences were considered significant at *P < 0.05, **P < 0.01, ***P < 0.001 and ****P < 0.0001, ns represents no statistical significance.

The melt and solvent printed scaffolds show typical compressive stress–strain curves of cellular polymeric structures, with an initial linear region exhibiting elastic behavior, followed by a switch from elastic to plastic region as the stress exceeds the yield point (**Figure** **2**a). The melt‐printed scaffolds present a steep linear elastic region with a higher maximum yield stress than the solvent‐printed scaffolds which have a shallow linear region and lower yield stress. This is confirmed by the compressive modulus and yield strength values (Figure 2d,e). The melt‐printed scaffolds present statistically higher compressive modulus, MPCL ≈43 MPa, than the solvent‐printed scaffolds, SPCL ≈10 MPa. Moreover, in both melt and solvent‐printed scaffolds it is possible to observe that the increase in the TCP loading increases the compressive modulus, ≈70 MPa and ≈22 MPa for MTCP40 and STCP40, respectively. The yield strength of melt‐extruded scaffolds is significantly higher than solvent printed scaffolds. Furthermore, the yield strength decreases by increasing the TCP concentration in the melt‐printed scaffolds, but this effect is not observed in the solvent scaffolds.

Dynamic cyclic compression results show the maximum compressive stress at 3% strain against the minimum recovered strain value at the stress of 0 MPa for each cycle (Figure 2b and Figure S5, Supporting Information). Melt‐printed scaffolds, within 10 cycles, show higher maximum compressive stresses at the strain of 3% and lower minimum recovered strains when the stress is removed, indicating a better tenacity and elasticity than the solvent‐printed scaffolds. Furthermore, for all samples, the maximum stress decreases, and the minimum recovered strain increases by increasing the number of cycles. In the case of the melt‐printed scaffolds, the addition of TCP particles increases the maximum stresses and slightly decreases the minimum recovered strain, indicating a stronger mechanical strength and elastic behavior. However, for solvent‐printed scaffolds, the addition of 20 wt% TCP particles improves the strength and elasticity of the scaffold but the strength against deformation and recoverability slightly decreases by increasing the TCP loading to 40 wt%, indicating that the high TCP concentration might weaken the mechanical properties of the solvent printed scaffolds. This trend can be also observed in Figure 2a,e, showing a flattened stress–strain curve and decreased yield strength, respectively. This might be attributed to microcracks formed in the filament.^[^ ^34^ ^]^ These microcracks are generated after the first loading cycle and grow larger by repeating the cycles. It can be also seen that the melt‐printed scaffolds show more narrow range of maximum stress and minimum recovered strain values than the solvent‐printed scaffolds, further confirming that the melt‐printed scaffolds have better ability against the deformation.

A time‐dependent behavior can be observed for all scaffolds and stresses decrease over time, indicating viscoelastic properties (Figure 2c). However, the stress relaxation rate of solvent‐printed scaffolds is substantially faster than the melted printed scaffolds and the increase of TCP leads to an increase in the relaxation rate, with STCP40 scaffolds showing the fastest relaxation rate amongst all sample types. This might be attributed to faster energy absorption and dissipation.^[^ ^35^ ^]^ Stress relaxation characteristics are important for scaffold design determining potential implant failure.^[^ ^36^ ^]^ Moreover, several studies also found that stress relaxation is correlated to osteogenesis, with faster stress relaxation significantly improving mesenchymal stem cell (MSC) osteogenic differentiation.^[^ ^24^, ^25^, ^37^, ^38^, ^39^ ^]^

Surface elastic modulus and hardness were determined using nanoindentation (Figure 2f,g). Overall, the melt‐printed scaffolds have a higher surface hardness and reduced elastic modulus than solvent‐printed scaffolds. The addition of TCP seems to increase the surface hardness of melt‐printed scaffolds, but no statistically significant differences were observed (Figure 2f). Contrary, STCP20 scaffolds present a significantly higher hardness (≈0.053 GPa) than SPCL (≈0.028 GPa) and all other samples, but the hardness decreases by increasing the TCP content to 40wt% (≈0.025 GPa). The reduced elastic modulus of melt‐ printed scaffolds increases from 0.61 to 1.02 GPa, by increasing the TCP loading (Figure 2g). In the case of solvent‐printed scaffolds, the surface elastic modulus exhibit a similar trend to the one observed for the surface hardness. As observed, STCP20 scaffolds show the highest reduced elastic modulus (≈1.21 GPa), among all samples, but a significant reduction is observed for STCP40 (≈0.6 GPa).

### 2.2 Possible Mechanisms Inducing the Differences of Physiochemical Properties between Melt and Solvent Printed Scaffolds

As mentioned in the previous section, melt‐ and solvent‐printed scaffolds present remarkably different physical properties. However, scaffolds were fabricated using the same chemical compositions, structural dimensions, and analogous processing parameters. Therefore, it is of great interest to investigate the mechanism that may induce these differences.

### 2.3 Layer Adhesive Bond Strength and Filament Tensile Properties

**Figure 3:**
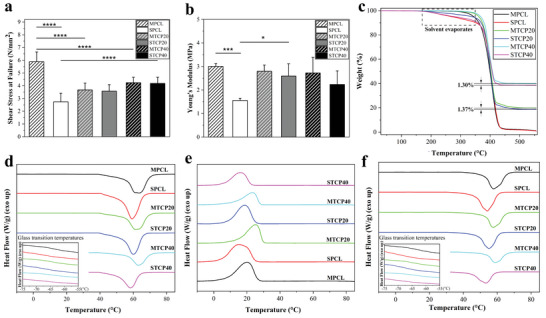
a) Shear stress between adjacent layers at failure (*n* = 5). b) Young's modulus of filaments (*n* = 5). Thermal properties of printed scaffolds. c) Representative TGA curves of all scaffolds. Representative DSC curves showing d) the first heating process and glass transition temperatures (inset image), e) the cooling process and f) the second heating process and glass transition temperatures (inset image) of all scaffolds. Data were presented as mean ± SD for a and b. Statistical analysis was performed using one‐way analysis of variance (ANOVA) for a and b. Differences were considered significant at *P < 0.05, **P < 0.01, ***P < 0.001 and ****P < 0.0001.

The adhesive bond strength between two adjacent layers within the scaffold was investigated using shear testing. As observed, MPCL scaffolds show the highest shear stress at failure (≈5.8 N mm^−2^), suggesting that these scaffolds have a better bond strength between layers than all the other samples (**Figure** **3**a). However, the presence of TCP significantly decreases the shear stress at failure for melt printed scaffolds, which might be ascribed to the fact that the addition of TCP accelerates the transition from the viscous “liquid‐like” state into the elastic “solid‐like” state, resulting in a poor layer bonding during the printing process.^[^ ^29^, ^40^ ^]^ In the case of solvent‐printed scaffolds, results show that the shear stress at failure increases by increasing the TCP content, with SPCL scaffolds exhibiting the lowest value among all samples (≈2.6 N mm^−2^). This might be caused by the irregular filament shape of SPCL scaffolds (Figure 1a), leading to a poor contact interface between layers and insufficient bonding strength. However, with the printing stability being enhanced by the incorporation of TCP particles, the printing quality of the filaments improve, strengthening the interfacial adhesion between adjacent layers. Contrary to MPCL and SPCL, no significant differences are observed between MTCP20, STCP20, MTCP40, and STCP40. Moreover, the Young's modulus of filaments follows a similar trend with a significant difference between MPCL and SPCL, with SPCL presenting the lowest modulus (Figure 3b). The MTCP filaments present a similar Young's modulus value to MPCL, but STCP filaments have higher Young's modulus than SPCL fila. These results might be also attributed to the irregular morphology of the filaments observed for SPCL scaffolds, which tends to disappear by adding TCP leading to a more circular‐shape filament. No significant differences were observed between all the other samples. These results seem to indicate that layer bond and filament tensile characteristics are strongly correlated to the printing stability and thus the morphological properties of the filaments. However, the results seem to indicate that the structural properties are not the governing factors to induce the distinctly different mechanical properties between melt and solvent‐printed scaffolds.

### 2.4 Thermal Characteristics

The thermal gravimetric analysis (TGA) results show that MTCP20 and MTCP40 scaffolds closely match the designed concentrations of TCP, ≈19.9% and ≈40%, respectively (Figure 3c and Table S2, Supporting Information). However, for solvent‐ printed scaffolds, both STCP20 and STCP40 scaffolds exhibit a lower TCP loading than the designed concentration, ≈18.6% and ≈38.6%, respectively. Moreover, although all the samples share a similar end‐point degradation temperature, the onset of degradation for solvent‐printed scaffolds occurs faster and at a lower temperature than the melt‐printed scaffolds. As observed, the degradation of solvent‐printed scaffolds undergoes two stages, an initial gradually decrease of weight (200 °C to 350 °C) followed by a sharp decrease (≈375 °C), whilst the melt‐printed scaffolds only show a sharp decrease. It is noted that the boiling point of both dibutyl phthalate (DBP) and 2‐butoxyethanol (2‐Bu) solvents are within the temperature range of the initial gradual degradation stage. After the gradual decrease of weight, the TGA curves of solvent‐printed scaffolds closely overlap those of melted printed scaffolds. Despite of the samples being left in a fume hood to evaporate the solvent prior to the experimental work, the results suggest the presence of residual solvent.

The differential scanning calorimetry (DSC) thermal characteristics of the scaffolds obtained from the first heating cycle represent the direct effects of the fabrication process (melt or solvent extrusion) on the melt temperature (*T*_m_) (Figure 3d,f, and Table S2, Supporting Information), whilst the second heating cycle is strongly dependent on the material properties. As observed, the solvent‐printed scaffolds present a lower *T*_m_ in both the first (≈58 °C) and second heating (≈54 °C) cycles, while melt‐printed scaffolds present higher melt temperatures (≈61°C and ≈57 °C, respectively). Results show that more energy absorption is required to destroy the crystal microstructure (first heating cycle) and to transform the polymer into an amorphous molten material, suggesting that the two printing techniques promote crystallization. These results are confirmed by the overall crystallinity, with all samples sharing a similar overall crystallinity (*X*_c_), ≈51–56%, in the first heating cycle, which decreases in the second heating cycle, ≈41–42% (Table S2, Supporting Information). No significant differences in *X*_c_ were observed between melt and solvent‐printed samples. Moreover, the melt‐printed samples present higher crystallization temperatures (*T*_c_) than solvent samples, indicating that they crystallize faster during cooling from the melt state (Figure 3e). These results indicate that, due to the presence of residual solvent, polymer chains in the solvent‐printed scaffolds present high mobility, lowering *T*_c_ and accelerating the transition from the solid semicrystalline state into the amorphous state (lower *T*_m_) in comparison to the melt‐printed samples. However, it is important to emphasize that the crystallization temperature of solvent printed samples is not obtained from the actual solvent state rather the melt state, thus subsequently does not represent the real crystallization kinetics during the printing process. For all samples, the glass transition temperatures in both heating cycles are similar, indicating that the fabrication process does not influence *T*_g_ (Figure 3d,f and Table S2, Supporting Information). Moreover, the addition of TCP particles has also limited impact on the thermodynamic properties of the composites. Nevertheless, the mechanism explaining the differences of physical properties between melt and solvent scaffolds is still unclear.

### 2.5 Crystal Microstructure

**Figure 4:**
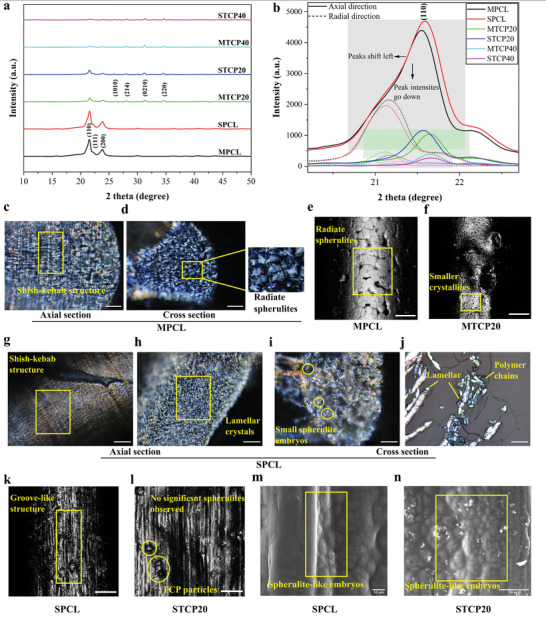
Crystal microstructure analysis. a) XRD pattern of melt and solvent printed scaffolds with typical PCL and TCP crystal planes. b) XRD pattern of all scaffolds showing typical PCL crystal plane (110) at the axial and radial direction, respectively. Polarized microscopy images of MPCL at c) axial section and d) cross section (scale bar: 30 µm). Reflectance confocal images of e) MPCL and f) MTCP20 (scale bar: 50 µm). Polarized microscopy images of SPCL at g,h) axial section and i,j) cross section (scale bar: 30 µm). Reflectance confocal images of k) SPCL and l) STCP20 (scale bar: 30 µm). SEM images of m) SPCL (scale bar: 10 µm) and n) STCP20 (scale bar: 50 µm).

The crystal microstructure of the printed scaffolds was assessed using X‐ray diffraction (XRD), as shown in **Figure** **4**a,b, and **Table** **1**. The typical crystal planes of PCL (110), (111), and (200) can be found at a 2‐theta ranging from 21° to 24°, indicating no chemical changes during the printing process. The reflection peaks of the PCL crystalline regions become flattened by increasing the TCP concentration, while the TCP peaks (1010, 214, 0210, and 220) increase, due to the reduction of PCL content and increase in TCP particle concentration.

**Table 1:** The values of 2 theta and crystallite size of scaffold samples at typical PCL crystal planes

	Crystal plane (110)		Crystal plane (200)	
	2 theta [°]	Crystallite size [nm]	2 theta [°]	Crystallite size [nm]
MPCL	21.48	12.49	23.82	12.06
SPCL	21.52	11.50	23.84	11.09
MTCP20	21.64	29.04	23.95	15.02
STCP20	21.55	18.20	23.87	13.39
MTCP40	21.65	32.88	23.95	24.21
STCP40	21.65	25.15	23.95	23.90

Table 1 shows the crystallite size obtained from the crystal planes (110) and (200). As observed, the crystallite sizes of all melt‐printed scaffolds are larger than the ones observed in the solvent‐printed scaffolds, suggesting greater lamellar crystallite formation in the melt‐printing scaffolds. Moreover, crystallite sizes for both solvent and melt printed scaffolds increase by increasing TCP concentration, implying the stacks or aggregates of lamellar crystallites grow due to the confinement effects of TCP particles.

The peak intensity as a function of printing direction was observed in the largest crystal plane (110) for PCL in both the axial direction (printing direction) and the radial direction (cross‐section) (Figure 4b). For all samples, the peak intensity significantly decreases in the radial direction indicating a highly aligned crystalline structure in the printing direction due to the high shear stress and confinement formed during the extrusion process.

Polymer crystallization is associated with the nucleation of fibrillar crystals and lamellae growth.^[^ ^41^, ^42^ ^]^ The aligned and folded polymer chains grow into a high ordered lamellar structure from the nucleation site. The crystals continue to grow by depositing polymer chains onto the growth front and eventually aggregate into spherulites. These processes change the density, symmetry, and phase transition of polymer and thus control the end properties of polymer products. Figure 4c–n shows the polarized microscopy, confocal reflectance, and scanning electron microscopy (SEM) images for both melt and solvent‐printed scaffolds. As observed, crystal morphologies are significantly different between the samples due to their different crystallization kinetics. Large and radiate spherulites can be found in MPCL scaffolds (Figure 4d,e), while small and fragmented lamellar crystals are observed in SPCL scaffolds (Figure 4h,j). Some spherulite‐like embryos can be found in SPCL polarized microscopy and SEM images (Figure 4i,m). However, unlike fibrillar crystals that predominantly grow in the radial direction in an ordered manner in MPCL scaffolds, the fibrillar‐like crystals in both SPCL and STCP20 scaffolds are randomly distributed around the nucleation sites and not able to stack into a bundle of lamellae and eventually forming spherulites (Figure 4k,l).

In the process of temperature‐induced crystallization (TIC), one of the critical conditions to form a spherulite is the large degree of undercooling/supercooling Δ*T* which provides large driving forces for crystallization (for melt printed scaffolds: Δ*T* = *T*_m_‐*T*_c_, the difference between the melting temperature and crystallization temperature for melt crystallization; for solvent printed scaffolds: Δ*T* = *T*_d_ ‐ *T*_c_, the difference between the dissolution temperature and crystallization temperature for solvent crystallization).^[^ ^42^, ^43^ ^]^ Complimenting the DSC results, the melt‐printed scaffolds crystallize from high temperature to room temperature, whilst the solvent‐printed scaffolds crystallize at room temperature, indicating a large difference of undercooling level and eventually leading to different microstructure kinetics and spherulite formation.

Moreover, the different spherulitic morphology may also be correlated to strain induced crystallization (SIC) during polymer confinement and flow within the printing nozzle. In Figure 4c,g, a shish‐kebab structure can be found in both melt and solvent‐ printed scaffolds, which is a typical morphology observed during SIC. The formation of a shish‐kebab structure is strongly dependent on the flow rate of the polymer during the processing stage.^[^ ^44^ ^]^ For the melt‐printed scaffolds, the high viscosity restrains the polymer mobility and leads to a relatively weaker flow and lower strain. Therefore, a shish‐kebab structure is not as obvious in the MPCL scaffolds (Figure 4c). Correspondingly, the MPCL scaffolds present an apparent spherulite morphology as shown in Figure 4d,e. However, in the case of solvent‐printed scaffolds, the motion of the polymer chains is significantly promoted due to the lower viscosity of the SPCL solution and the presence of solvent. The strong flow and high strain lead to the formation of an apparent shish‐kebab structure (Figure 4g) and groove or flake‐like features on the filament surfaces (Figure 4k).

The addition of TCP particles leads to smaller spherulite regions and large number of spherulites (Figure 4e,f,m,n). This can be attributed to the confinement effect which restrains the spatial growth of spherulites.

Herein, two crystal growth kinetics may play a governing role that affects the apparent physio‐chemical properties of both melt and solvent‐printed scaffolds.

### 2.6 Biological Response to Melt and Solvent‐Printed Scaffolds

To assess the cellular response to the two different types of scaffolds, human adipose‐derived stem cells (hADSCs) were seeded and cell metabolic activity, proliferation, morphology, and osteogenic differentiation were analyzed.

**Figure 5:**
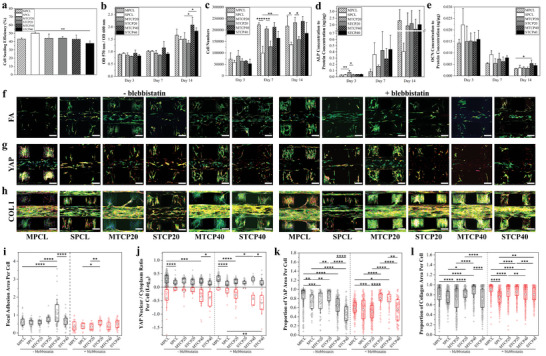
Biological responses of printed scaffolds. a) Cell seeding efficiency of melt and solvent printed scaffolds (n = 4). b) Metabolic activity at day 3, 7, and 14 (*n* = 4). c) MTT results presenting the cell number at days 3, 7, and 14 (*n* = 4). d) Normalized ALP expression (*n* = 4) and e) normalized OCN expression of all scaffolds showing osteogenic differentiation of hADSCs at days 3, 7, and 14 (*n* = 4). Confocal images showing f) focal adhesion expression, g) YAP localization, and h) collagen type I expression for melt and solvent printed scaffolds with (+) and without (‐) blebbistatin treatment (scale bar: 200 µm; blue = nuclei, green = actin, and red = FA, YAP, COL I, respectively). Quantification of i) focal adhesion area per cell (*n* ≥ 25), j) YAP nuclear localization in logarithm values (*n* ≥ 33), k) proportional YAP area (*n* ≥ 33), and l) proportional collagen type I (*n* ≥ 245). (Box plot presents 25^th^ to 75^th^ percentiles, mean and standard deviation (coef = 1)). Data were presented as mean ± SD from a to e and i to l. Statistical analysis was performed using one‐way analysis of variance (ANOVA) for a to e and i to l. Differences were considered significant at *P < 0.05, **P < 0.01, ***P < 0.001 and ****P < 0.0001.

For all scaffolds, a cell seeding efficiency of around 40–50% was achieved, with SPCL scaffolds exhibiting a significantly higher efficiency than STCP40 and STCP40, which presents the lowest initial cell attachment (**Figure** **5**a). The slightly higher cell attachment observed for SPCL scaffolds may be attributed to the groove‐like surface morphology which can trap cells during the static seeding process. Cell metabolic activity presents an increasing trend with cell culture time, indicating no significant cytotoxicity (Figure 5b). At day 14, a significant increase in the absorbance values was observed for all scaffolds in comparison to day 1, demonstrating significant cell growth and increased metabolic activity. Both MTCP40 and STCP40 scaffolds exhibit higher absorbance values suggesting that the addition of TCP enhances cell metabolic activity. Moreover, results seem to indicate higher absorbance values for melt‐printed scaffolds, but no statistical differences were observed between melt and solvent‐printed scaffolds for the same material composition. The 3‐(4,5‐dimethylthiazol‐2‐yl)‐2,5‐diphenyl‐2H‐tetrazolium bromide (MTT) assay was used to further investigate cell proliferation on the scaffolds (Figure 5c). At an early culture stage (day 3), no significant differences were observed in the number of cells for all scaffolds. However, at days 7 and 14, melt‐printed scaffolds present significantly higher cell numbers than solvent‐printed scaffolds. Additionally, there is an increasing trend in cell proliferation with increasing TCP concentration, especially for solvent‐printed scaffolds. However, no statistical differences were observed between PCL only and TCP containing scaffolds.

The osteogenic differentiation of hADSCs seeded on melt and solvent‐printed scaffolds was assessed through the expression of alkaline phosphatase (ALP) and osteocalcin (OCN) at days 3, 7, and 14. ALP results show that the normalized concentration of ALP significantly increases from days 3 to 14, indicating a promoted osteogenic differentiation of hADSCs in both melt and solvent scaffolds (Figure 5d). At day 3, MTCP20 scaffolds show a greater normalized ALP concentration than MPCL scaffolds, but no significant differences were observed between other groups. At day 7, solvent‐printed scaffolds show a relatively higher ALP activity than the melt‐printed scaffolds, and the ALP expression increases by increasing the TCP concentration in both melt and solvent‐printed scaffolds. However, no statistical differences were observed between samples. A significant increase in ALP is observed at day 14 compared to day 7 for all scaffold groups, except SPCL, but no statistical differences were observed between the samples. OCN is recognized as a late‐stage maker of bone formation, but its specific role in osteogenesis is still not clear.^[^ ^45^ ^]^ However, OCN can still be a useful marker to assess differentiation in the melt and solvent‐printed scaffolds. As observed, OCN expression is similar for all samples at each timepoint and unexpectedly there is a gradually decrease with the differentiation culture time (Figure 5e). Solvent‐printed scaffolds present relatively higher OCN concentrations than melt‐printed samples. As observed, at days 3 and 7, SPCL scaffolds exhibit the highest normalized OCN concentration, but no statistical differences were observed between the different groups. At day 14, MTCP40 scaffolds show statistically higher OCN values than MPCL scaffolds, but no significant differences were observed between other scaffold groups.

Cell proliferation and differentiation in the scaffolds are closely correlated with the fabrication technique, rather than with the TCP concentration (chemical cues) which follows a similar trend in cell behavior changes regardless of fabrication technique (Figure 5a–e). Results suggest that physical cell‐scaffold interactions may play a vital role in regulating cellular response. Among them, the mechanical stimuli of the scaffold and cell sensing via mechanotransduction pathways are of great interest. Confocal fluorescence microscopy and statistical image analysis (Figure 5f,i) show that melt‐printed scaffolds show an overall higher expression of focal adhesions (FAs) than the solvent‐printed scaffolds except for STCP20 scaffolds. Moreover, MTCP40 scaffolds present the highest expression of FA than the other groups. When the blebbistatin, a myosin inhibitor, is used to block cytoskeleton tension, the expression of FAs for all scaffolds is downregulated. Among all considered groups, STCP20 scaffolds show the highest expression of FAs. These results indicate that the FA expression is closely linked with the surface hardness of the scaffolds.

Yes‐associated protein (YAP), a transcriptional regulator of mechanotransduction, was used to further investigate the cellular responses towards the surface stiffness of the scaffold's filaments. It is recognized that YAP is localized in the cytoplasm when cells are experiencing low levels of mechanical stimuli or small adhesion areas, while they are in the nuclei when perceiving intensive mechanical signals.^[^ ^46^ ^]^ Therefore, the ratio of nuclear YAP/cytoplasm YAP was used to identify the substrate rigidity. Figure 5g,j shows the confocal images of YAP staining and the logarithm values of the ratio between nuclear and cytoplasm YAP, with positive values indicating that the YAP located in the nuclei is higher than in the cytoplasm. As shown, for all scaffolds most of YAP are localized in the nuclei, indicating an increased cellular tension. However, the melt printed scaffolds show higher expression of nuclear YAP than the solvent‐printed scaffolds. Among all scaffold groups, MPCL scaffolds show the highest nuclear YAP, while STCP40 scaffolds show the lowest values. Contrary to the positive YAP ratios, no statistically significant differences between samples were observed for the negative ratios for the noninhibited case. A similar trend was also observed for scaffolds treated with the inhibitor, with SPCL and STCP40 scaffolds showing relatively lower nuclear YAP. Moreover, STCP40 inhibited scaffolds show statistically higher cytoplasm YAP than SPCL. These results show that hADSCs perceive the stiffer surface of melt printed scaffolds and translate these mechanical cues to regulate YAP allocated in the nuclei and this trend is not altered by using the myosin inhibitor. The expression of YAP also follows this trend to some extent (Figure 5k). STCP20 scaffolds show statistically higher expression of YAP per cell than all the other groups, while STCP40 scaffolds show statistically lower values than all the other groups. As observed, the expression of YAP in the MPCL, SPCL and MTCP20 scaffolds is downregulated when these scaffolds are treated with the inhibitor and STCP20, MTCP40 and STCP40 inhibited scaffolds show similar values in terms of YAP expression per cell. These results suggest that stiffer surfaces promote the expression of YAP. However, how the prevailing nuclear YAP after the inhibition and whether TCP as a chemical cue influence the expression of YAP are still unknown.

The effects of mechanotransduction on cellular activities, specifically collagen type I expression (COL‐I), are shown in Figure 5h,l. Results show that after 3 d of cell proliferation and cell differentiation, respectively, an intensive cell membrane is formed and spreading along the printed filaments, particularly in the case of melt printed scaffolds. Moreover, a large amount of COL‐I can be observed on all scaffolds, suggesting the existence of osteogenesis differentiation. The treatment with myosin inhibitor seems not to restrain cell growth (Figure 5f,g,h) and the secretion of COL‐I. The semiquantification of COL‐I indicated in Figure 5l shows that TCP40, STCP20, and MPCL scaffolds present statistically higher expression of COL‐I than SPCL, MTCP20, and STCP40 scaffolds. After the inhibitation, it can be observed that all scaffolds slightly promote the secretion of COL‐I in comparison to the non‐inhibited groups. Among all inhibited scaffolds, MPCL presents the highest value of COL‐I while SPCL shows significantly lower COL‐I expression. As reported, MSCs osteogenesis fate is adopted in stiff substrates, while soft substrates favor adipogenesis and chondrogenesis.^[^ ^47^, ^48^ ^]^ This might explain the increased COL‐I expression as it is also the product of both cell fates. Based on the results presented in Figure 5d,e, it can be assumed that the mechanotransduction (substrate stiffness) influences the osteogenesis differentiation on both melt and solvent‐printed scaffolds, while the addition of TCP presents limited synergetic effects.

## 3 Discussion

In this work, 3D bone scaffolds were fabricated using melt and solvent extrusion‐based AM techniques and their characteristics extensively investigated. In order to successfully print a scaffold, the printability of a biomaterial is a key concern and is typically associated with the material intrinsic nature (e.g., molecular weight), the filler concentration, processing conditions and rheological properties (e.g., viscosity, shear thinning). Among them, the polymer melt/solution flow is a critical criterion of printability.

The phase transition that occurs in a polymer melt with high loading of fillers restrains the flowability of the composite, and consequently the printability is reduced unless increased processing temperatures are used. Although Park et al.^[^ ^49^ ^]^ have fabricated 50/50 wt% and 70/30 wt% TCP/PCL scaffolds using melt printing, the produced scaffolds were brittle and show poor compressive strength compared to the PCL scaffold. Moreover, the high concentration of fillers, especially nanomaterials, can cause agglomeration and lead to an inhomogeneous distribution of fillers in the polymer matrix and needle‐clogging problems. Therefore, in this study high concentrations of TCP (>40 wt%) were not used.

In contrast, solvents used in polymer solutions promote the diffusion of polymer chains by reducing the glass transition temperature^[^ ^50^ ^]^ and increasing the free volume in the polymer matrix enhancing the packing capacity.^[^ ^51^ ^]^ This allows the use of high loading of fillers and nanomaterials. For instance, Jakus et al.^[^ ^9^ ^]^ reported the use of high‐loaded graphene polymer solution (75 wt%) to fabricate an electronic bioscaffold without compromising the mechanical properties. More importantly, unlike the high temperature used in melt printing, solvent printing is usually performed under mild temperature, allowing the use of low thermally stable materials such as natural polymers and biomolecules.^[^ ^8^, ^11^ ^]^ However, extrudate distortion is more likely to occur during solvent extrusion due to the lubricant effect of the solvents, thus causing wall slip, especially in unfilled polymer solutions.^[^ ^52^, ^53^ ^]^ As shown in Figure 1a, the melt‐printed scaffolds show more uniform and regular shape than the solvent‐printed PCL scaffolds, which present an irregular cross‐section and groove or flake‐like surface morphology. However, the extrudate distortion can be significantly improved through the addition of TCP particles, which may be attributed to the anchoring effect of TCP particles and less wall slip. These findings are in agreement with previously reported works suggesting that an increase on the filler concentration strongly reduces the extrudate distortion.^[^ ^52^, ^54^ ^]^

**Figure 6:**
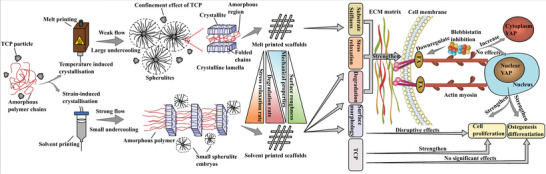
Schematic illustration. It shows the crystal growth mechanism on melt and solvent‐printed scaffolds and the mechanotransduction pathway from the scaffold substrate to the cell nucleus.

The melt‐printed and solvent‐printed scaffolds show distinct physical properties despite the same chemical composition, structural dimensions, and similar processing conditions were used. This can be attributed to the different crystal growth kinetics due to specific TIC and SIC processes, as shown in **Figure** **6**, resulting in distinct microstructures. TIC plays a dominant effect in the melt‐printed scaffolds, providing a large degree of undercooling, and leading to the formation of large and well‐organized spherulites (Figure 4d,e). The spherulites grow outwards radiatively from nucleating sites and stop growing due to the impingement of adjacent spherulites. This contributes to the formation of a typical spherulite‐like surface morphology. Moreover, TCP particles confine the spatial growth of spherulites leading to smaller spherulites and more intensive crystalline regions on the scaffold surface (Figure 4f). SIC mainly prevails in the solvent‐printed scaffolds. Unlike TIC, which relies on a temperature gradient and cooling resulting in a relatively slow process allowing crystals to grow, SIC is an immediate process.^[^ ^55^ ^]^ The low undercooling and short growing time prohibit the stacking of bundles of lamellae, whilst the high shear rate leads to the formation of a shish‐kebab crystal structure, causing the groove or flake‐like morphology on the solvent‐printed scaffolds (Figure 4k–l).

Wettability results show that the contact angle increases with increasing TCP concentration for melt‐printed scaffolds while a decrease is observed for solvent‐printed scaffolds (Figure 1d). A similar trend can be found in terms of the surface roughness (Figure 1c). TCP decreases roughness in melt‐printed scaffolds and increases roughness in solvent‐printed scaffolds. The curvature of the melt‐printed filaments is all similar with a circular profile. The solvent‐printed filament curvature evolves with TCP concentration, moving from an irregular flake‐like surface of SPCL to a more circular profile of STCP40 (Figure 1a). The MPCL and SPCL filaments show a quite similar surface roughness and the difference observed in wettability, although not significant, could be attributed to the irregular curvature profiles of SPCL, leading to the pinned droplets at the edge of grooved substrate surface.^[^ ^56^ ^]^ However, as the TCP concentration increases, the curvature of solvent‐printed filaments becomes more circular, resembling the melt‐printed filaments. Moreover, the surface roughness significantly increases in STCP20 and STCP40 corresponding with a trend of decreasing contact angle (more hydrophilic). Considering the similar scaffold dimensions, printed structures, and compositions for both printing techniques, it can be suggested that, instead of the filament curvature, the surface topography may play a dominant role in the wettability. As previously mentioned, the formation of micropores is mitigated with the increase of TCP, leading to the decreased surface roughness for melt‐printed scaffolds. The decreased micropores minimize the capillary effect or water absorption on the timescales observed, subsequently leading to an increase in water contact angle. Inversely, the solvent‐printed scaffolds present an increase in surface roughness and micropores particularly for STCP40. Therefore, the water contact angle decreases, and the solvent‐printed samples become more hydrophilic, accelerating water infiltration into the solvent‐printed scaffolds, as demonstrated with STCP40. The increase in surface roughness of a printed filament has been demonstrated to promote hydrophilicity in PCL scaffolds.^[^ ^57^, ^58^ ^]^

The two different crystal growth kinetics and microstructures impact in the degradation properties of the scaffolds. Hydrolytic degradation of polymers decreases by increasing crystallinity.^[^ ^59^ ^]^ However, the DSC results show that both melt and solvent‐printed scaffolds share a similar crystallinity. Therefore, results seem to suggest that the hydrolysis of the amorphous regions have a significant impact on the degradation rate. As illustrated in Figure 6, a highly ordered and compact spherulite structure is formed in the melt‐printed scaffolds with amorphous polymer chains within the lamellae. The intensive and ordered structures hinder the hydrolytic attack of the amorphous regions. Contrarily, the dispersed and fragmented lamellae in the solvent‐ printed scaffolds enable freer access to the amorphous polymer chains. Moreover, TCP is soluble thus as it dissolves at the PCL matrix surface, a greater interfacial surface area will be exposed to hydrolytic attack, subsequently accelerating the exposure of amorphous regions and leading to a faster degradation rate. A similar phenomenon was observed by Lam et al.^[^ ^31^ ^]^ Additionally, the rapid degradation of STCP40 can also be attributed to the surface superhydrophilicity which accelerates diffusion into the polymer matrix.^[^ ^31^, ^32^ ^]^

Furthermore, the distinct mechanical properties observed in Figures 2 and 3a,b advance the notion of specific crystal growth kinetics and microstructures in the melt and solvent‐printed scaffolds. The higher compressive modulus of melt printed scaffolds and the increase of compressive modulus by increasing the TCP concentration that was observed in both scaffold types, suggests that the compact crystal structures and TCP particles induce a better resistance against compressive force. Moreover, the confinement of TCP particles which leads to the aggregation of PCL lamellae and larger crystallite size further contributes to the dissipation of compressive stress.

However, the spherulite growth is an individual and isolated process. The lack of interfacial adherence between spherulites and the poor PCL‐TCP interactions lead to similar filament Young's modulus and tensile strengths between melt and solvent printed scaffolds, except for SPCL scaffolds due to the irregular shape (Figure 3b and Figures S6–S8, Supporting Information). Reports suggested that solid surfaces in a polymer matrix serve as a heterogeneous nucleation agent and induce transcrystallization.^[^ ^60^ ^]^ For instance, high aspect ratio carbon nanotubes have been reported to induce a dense transcrystalline layer in a polymer matrix resulting in a high modulus and tensile strength.^[^ ^61^, ^62^ ^]^ However, in this work, the interaction between PCL and TCP is not evident (Figure 4c,d,g–j). This might be attributed to the microscale particle size which is unable to provide a large interfacial surface area.

Cellular behavior alters in response to nanomechanical stimuli (e.g., surface hardness and stiffness) via mechanotransduction pathways. Overall, the melt‐printed scaffolds show higher surface hardness and stiffness than solvent‐printed scaffolds. We ascribe this to the denser crystalline chain packing. Interestingly, STCP20 also presents high surface hardness and stiffness. This may be a contribution to the less volume of solvents used during the material preparation process which restrains the polymer chain mobility. However, further investigation is required to understand the effects of solvent volumes on the crystallization process.

Stem cell fate is strongly determined by the physical features of the cell microenvironment in addition to soluble signals.^[^ ^17^, ^63^, ^64^ ^]^ Therefore, scaffold properties such as topography, degradability, stress‐relaxation, and substrate stiffness play a major role on the influence of hADSCs proliferation and their phenotypic lineage commitment (e.g., osteogenic differentiation). Through mechanotransduction pathway, cells can convert mechanical signals into biological responses. Integrins attach to extracellular matrix (ECM) and sense the stimulus, followed by the maturation of FA that bind to actin‐myosin under the sufficient stimulus.^[^ ^39^ ^]^ A trans‐membrane connection is formed between the ECM and the cytoskeleton, allowing physically linking the ECM and intracellular mechanotransduction elements.^[^ ^65^ ^]^ The linker of the nucleoskeleton and cytoskeleton complex allows the actin‐myosin to bind to the nucleus. The actin‐myosin filaments form contractile bundles, which can exert forces on the FAs and create cellular tension. YAP and transcriptional coactivator with PDZ‐binding motif (TAZ), the most influential mechanosensitive transcription factors, translocate to the nucleus owing to the cytoskeletal tension, directly regulating gene expression and eventually mediating the biological responses.^[^ ^66^ ^]^

A classic model of controlling cell proliferation is the contact inhibition of proliferation (CIP), where cultured cells stop growing at a critical cell density.^[^ ^66^ ^]^ This is ascribed to the inhibition of YAP/TAZ, with cell crowding confining growth space, leading to a low contractility and repressive cytoskeletons.^[^ ^67^ ^]^ However, this can be overridden by stiffening the ECM, loss of actin capping and severing proteins and stretching, allowing the activation of YAP/TAZ transcription.^[^ ^67^, ^68^, ^69^ ^]^

In this study, both melt‐ and solvent‐printed scaffolds present a high nuclear allocation of YAP, indicating sufficient cellular tension due to the high stiffness of the scaffold surface. However, melt‐printed scaffolds, with a stiffer surface, have a higher ratio of nuclear YAP than solvent‐printed scaffolds (Figure 5j). As a result, the higher hADSCs proliferation for the melt‐printed scaffolds (Figure 5c) suggests the overcoming of CIP. Interestingly, STCP20 with a stiffer surface still presents a relatively lower proliferation rate. This is likely due to the surface topography, as the shape and irregular surfaces also affect the cell morphology on chromosomal positioning and gene regulation,^[^ ^70^ ^]^ and regulate mechanotransduction, hence cellular migration and proliferation.^[^ ^71^ ^]^ Due to the SIC, the groove or flake‐like surface topography on the solvent‐printed scaffolds guides and restricts the hADSCs spreading along the filament, creating a dense and elongated cell morphology, as shown in Figure 5f,g,h. This may generate a localized high cell density inhibiting cellular contractility. The protrusions of the groove or flake‐like topography also disrupt the gap junctions as a physical barrier, hindering cell–cell communications. In addition, cell metabolic activity and proliferation results show the positive effects of TCP, suggesting that other signaling pathways may also be involved in the regulation of hADSC proliferation, which requires further investigation (Figure 5a–c).

Mechanotransduction pathways regulate the osteogenesis behavior differently in 2D and 3D environments. In 2D, cells are able to build up high cellular tension with a large number of FAs, actin stress fibers, and nuclear YAP on stiff substrates.^[^ ^19^, ^72^ ^]^ In turn, this upregulation of mechanotransduction significantly promotes osteogenesis differentiation pathways and cell proliferation. Moreover, this upregulation seems to not have an upper limit to substrate stiffness in 2D cell culture. However, it is considered less relevant in a 3D environment.^[^ ^39^ ^]^ This is ascribed to the abundant and close proximity of adhesive ligands that can be recruited in 2D, allowing easy integrin clustering and FAs formation to build up cellular tension. Contrary, in a 3D environment, these ligands are spatial distributed, embedded in a matrix, and required to gather before integrin clustering.^[^ ^39^ ^]^ Therefore, the matrix remodeling is a key factor for the mechanotransduction in a 3D cell microenvironment, being less relevant in 2D environments.^[^ ^22^ ^]^ Thus, engineering 3D biomaterials or scaffolds require dynamic surfaces (e.g., degradation and stress relaxation) to facilitate the matrix remodeling, instead of a quiescent state in a nondynamic 2D substrate. In this study, both melt and solvent printed scaffolds show nuclear YAP allocation, with the melt printed scaffolds exhibiting higher ratio of YAP in nuclei. These results suggest that both types of scaffolds present suitable surface stiffness with the melt‐printed scaffolds presenting the better surface stiffness characteristics. However, in the early stages of osteogenesis, solvent‐printed scaffolds show relatively higher osteogenic marker expression (ALP and OCN at day 7) than the melt‐printed scaffolds. In combination with the results shown in Figures 1b and 2c, we presume that the faster degradation and stress relaxation properties of the solvent‐printed scaffolds contribute to the ECM matrix remodeling, enhancing mechanotransduction pathways and contributing to osteogenic differentiation. Nevertheless, due to the degradation process and stress relaxation along time, melt‐printed scaffolds also tend to provide a dynamic surface as the solvent‐printed scaffolds, which together with a stiffer surface explains the slightly higher ALP and OCN expressions observed for melt‐printed scaffolds in comparison to solvent printed scaffolds.

As observed, the mechanotransduction pathway regulation of osteogenic differentiation in the 3D printed scaffolds is not only dependent on the substrate stiffness but also relies on the dynamic surfaces of the engineered scaffolds. Among them, the mechanism of how dynamic surface modulating ECM matrix and whether other signaling pathways (i.e., stimulated through the addition of TCP) are involved in the regulation of osteogenesis requires further investigation.

## 4 Conclusion

Melt and solvent extrusion AM were used for the fabrication of PCL and PCL/TCP scaffolds for bone tissue engineering applications. Although extrusion is a widely used AM technique, solvent extrusion to directly create 3D porous scaffolds is a relatively new approach. Moreover, there is limited understanding of the differences arising from using either melt or solvent extrusion on the characteristics of printed scaffolds. This study provides a comprehensive characterization of both melt‐ and solvent‐printed scaffolds and the resulting process–structure–property relationships.

Despite sharing similar dimensional characteristics (scaffold design), chemical composition and processing conditions, melt and solvent‐printed scaffolds exhibit distinct physical properties including mechanical, scaffold wettability, topography, and degradability properties. Both approaches allowed the fabrication of well‐defined 3D porous composite scaffolds, but the crystal growth kinetics and microstructure significantly differ, explaining the different physical and biological properties between these two types of scaffolds.

The polymer in the melt printing process undergoes TIC, providing a large degree of undercooling that enables growth kinetics allowing the formation of large and well‐organized spherulites, and leading to better mechanical properties, stiffer substrate, and spherulite impingement morphology. On the other side, solvent printing is dominated by SIC resulting in the formation of small and fragmented lamellae and shish‐kebab crystal structures due to the rapid crystallization and high shear rate. Subsequently, faster degradation and higher stress relaxation are observed. The TCP particles have a confinement effect within the crystal structures, resulting in small size spherulites and a more intense and aggregated crystalline region. The differences in material flow and crystallization impact the surface topography, with the solvent‐printed scaffolds presenting a groove or flake‐like appearance that becomes smoother with increasing TCP concentration, while all melt scaffolds are smooth.

These distinct characteristics influence cellular proliferation and osteogenic differentiation due to the intimate cell‐scaffold interactions and mechanical stimuli that modulate mechanotransduction pathways. The higher stiffness of the melt‐printed scaffolds contributes to greater cell proliferation than solvent‐printed scaffolds. The addition of TCP, as a biochemical cue, enhances cell proliferation. Moreover, the higher ratio of nuclear YAP as a result of the stiffer substrate of melt‐printed scaffold favors osteogenesis, concurrently, the faster degradation and stress relaxation of the solvent‐printed scaffolds also contribute to an early stage of osteogenic stem cell fate.

This study demonstrates the importance of AM processing route selection for bone tissue engineering applications, as scaffolds with distinctive microstructures have been fabricated from the same material constituents. This allows scaffolds to be engineered for specific applications.

## 5 Experimental Section

### Materials

PCL (CAPA 6500, *M*_w_ = 50 kDa) was obtained from PerstorpCaprolactones (UK). TCP (*M*_w_ = 310.8 g mol^−1^, Puriss ≥ 98%), dichloromethane (DCM), DBP, 2‐Bu, fetal bovine serum (FBS), phosphate‐buffered saline (PBS), hexamethyldisilazane (HDMS), 10% neutral buffered formalin, Triton X‐100, radioimmunoprecipitation assay (RIPA) buffer, anti‐collagen type I primary antibody, protease inhibitor cocktail, and Alamar Blue were all purchased from Sigma‐Aldrich (UK). hADSCs, MesenPRO RS basal media, STEMPRO osteogenesis differentiation kit, Alexa Fluor 488‐conjugated phalloidin, 4′,6‐diamidino‐2‐phenylindole (DAPI), anti‐collagen type I primary antibody, goat anti‐mouse IgG secondary antibody (Alexa Fluor Plus 647), and the bicinchoninic acid assay (BCA, Micro BCA Protein Assay Kit) were obtained from Thermo Fisher Scientific (UK). SensoLytepNPP ALP assay kit was purchased from AnaSpec (USA). MTT cell proliferation, OCN assays, anti‐YAP primary antibody, recombinant Alexa Fluor 647 anti‐paxillin antibody, and blebbistatin were obtained from Abcam (UK).

### Scaffold Fabrication

To perform both melt extrusion and solvent extrusion 3D printing technologies, composite materials were separately prepared using melt blending and the solvent blending techniques. The considered composite materials were named as shown in **Table** **2**. For melt blending, PCL pellets were heated up to 90 °C for 20 min followed by the addition of TCP powders into the melted PCL matrix. The blends were physically mixed for at least 30 min to achieve a homogeneous dispersion of TCP powders. After cooling down to room temperature, the composite materials were cut into small pieces and ready for the scaffold fabrication.

**Table 2:** Scaffold type, ceramic content, and material preparation methods

Sample	Abbreviation	Processing	PCL [wt%]	TCP [wt%]
PCL	MPCL	Melt printing	100	0
PCL	SPCL	Solvent printing	100	0
PCL/TCP (80/20 wt%)	MTCP20	Melt printing	80	20
PCL/TCP (80/20 wt%)	STCP20	Solvent printing	80	20
PCL/TCP (60/40 wt%)	MTCP40	Melt printing	60	40
PCL/TCP (60/40 wt%)	STCP40	Solvent printing	60	40

The solvent blending was performed based on the protocol described by Jakus et al.^[^ ^7^, ^73^ ^]^ Briefly, the composite inks were prepared by dissolving PCL and TCP powders in a mixture of 2‐Bu, DBP, and excess of DCM solution. 0.6 g DBP was added for every 1 cm^3^ TCP powders and the mass ratio of DCM: 2‐Bu: DBP was 20:2:1. The dissolved PCL and TCP suspensions were physically mixed by an overhead stirrer, allowing to thicken via evaporation of DCM in a fume hood at room temperature. The evaporation process can take up to several hours until the appropriate viscosity is reached for 3D printing (30–35 Pa s).^[^ ^8^ ^]^ Prepared inks were immediately stored at 4 °C in glass jars with a proper sealing and ready for further use.

A screw‐assisted extrusion‐based AM (3D Discovery, RegenHU, Switzerland) was used to fabricate the melt‐extruded scaffolds, considering a 0°/90° lay‐down pattern, filament width of 330 µm, 350 µm pore size, and the 270 µm layer thickness. During the printing process, the prepared materials were heated up to 90 °C and extruded out from a 330 µm inner diameter nozzle with a screw rotational velocity of 12 rpm, feed rate of 20 mm s^−1^ and air pressure of 6 bar. The scaffold dimensions were 30 mm × 30 mm × 2.5 mm for biological studies and 30 mm × 30 mm × 5 mm for mechanical studies.

Solvent extruded scaffolds were fabricated using a pneumatic‐based bioprinter (3D Bioplotter, EnvisionTEC, Germany) at room temperature. In order to obtain similar geometric architectures to the melt‐extruded scaffolds, the solvent extruded scaffolds were designed considering the same geometric parameters and printed with a feed rate of 20 mm s^−1^ and a 406 µm conical needle. Due to the change of ink viscosity caused by the DCM evaporation during the printing process, the air pressure varied between 3.5 bar and 5.0 bar to obtain a filament diameter close to the designed one as assessed by optical microscopy (Leica DMi1, Germany). The scaffold dimensions were 30 mm × 30 mm × 2.5 mm for biological studies and 30 mm × 30 mm × 5 mm for mechanical studies. All solvent‐printed scaffolds were stored in a fume hood for several weeks, allowing solvent evaporation.

### Scaffold Morphology and Porosity

SEM (Quanta 250, FEI Company, USA) was used to assess the morphological properties of the scaffolds. Scaffolds were sputtered coated (Q150T ES, Quorum Technologies, UK) with a 6 nm thick gold/palladium (80:20) coating. Both top and cross‐sections of the scaffolds were imaged and analyzed using the ImageJ software. Confocal reflectance microscopy (SP8, Leica Microsystems, Germany) was used to assess filament surface morphology. Scans at 488 nm with an *x*‐*y* and *z* pixel size of 0.124 nm and 2.409 µm, respectively, were collected. A maximum intensity z‐projection of the stack using the ImageJ software was applied to obtain the 2D images.

The porosity of the scaffolds (*n* = 5) was calculated using the gravimetric method according to the following equations:^[^ ^74^, ^75^ ^]^

(1) $$\mathrm{Porosity}\;\left(\%\right)=\left(1-\frac{\rho_{s}}{\rho_{m}}\right)\times 100$$

(2) $$\rho_{s}=\frac{m_{s}}{v_{s}}$$

(3) $$\rho_{m}=100/\left(\frac{w_{p}}{\rho_{p}}+\frac{w_{t}}{\rho_{t}}\right)$$

where *ρ*_s_ is the apparent density of the scaffold, *ρ*_m_ is the density of PCL/TCP composite material; *m_s_* is the measured mass of the scaffold, *v_s_* is the volume of the scaffold based on the dimensions measured by a caliper; *w*_p_ and *w*_t_ are the weight percentage measured by TGA analysis; *ρ*_p_ = 1.145 g cm^−3^ and *ρ*_t_ = 3.14 g cm^−3^ refer to theoretical densities of PCL and TCP respectively.

Surface topography was investigated using AFM (MultiMode V, Bruker, USA) and the surface roughness was measured as the arithmetic mean value *R*_a_, considering at least five measurements for each sample.

### Mechanical Analysis: Static and Dynamic Compression

Uniaxial static and dynamic compression tests were performed using the Instron 3344 (Instron, USA) system equipped with a 100 N load cell. Scaffolds were cut to approximately 4.5 mm (*L*) × 4.5 mm (*W*) × 5 mm (*H*) samples for both uniaxial and dynamic compression tests. Each test was performed six times. As the melt‐printed samples are more rigid than solvent‐printed samples, considering the limit of 100 N load cell, the static compression tests were performed at a strain rate of 0.5 mm min^−1^ up to strain of 10% for melt‐extruded and solvent‐extruded scaffolds. The compressive modulus was obtained from the linear region of the stress–strain curve and the yield strength was determined as the stress at which the constructed line (0.2% strain offset) intersected the strain–stress curve.

Dynamic cyclic compression tests were performed at a 0.5 mm min^−1^ ramp rate up to 3% strain with a preload of 0.5 N for 10 cycles. The cyclic stress‐strain curves were presented and the minimum recovered strain (stress = 0 MPa) and maximum compressive stress (strain = 3%) for each cycle were analyzed.

### Stress Relaxation

For stress relaxation analysis, the samples (*n* = 3) were compressed up to a strain of 10% at a strain rate of 0.5 mm min^−1^. The compressive stress was recorded over time with the strain being kept unchanged. The stress was normalized to the maximum stress and plotted against time.

### Filament Tensile Test

Uniaxial tests were performed to assess the individual filament tensile behavior using the Instron 3344 (Instron, USA) system, equipped with a 10 N load cell. Printed samples (*n* = 5, 40 mm in length) were mounted onto paper windows with double‐sided tape. Paper windows were gripped to tensile grips to allow a 10 mm gauge length, and a constant strain rate of 5 mm min^−1^ was applied until fracture occurred.^[^ ^76^ ^]^ The Young's modulus was obtained from the strain‐stress curve, using the slope of the linear region. The tensile strength (*σ*) and elongation at break (%*δ*) were calculated as follows:

(4) $$\sigma =\frac{F_{t}}{A_{t}}$$

(5) $$\%\delta =\frac{\left(L_{f}-L_{0}\right)}{L_{0}}\times 100$$

where *F*_t_ is the tensile force at break, *A*_t_ is the cross‐sectional area of the printed filaments; *L*_f_ refers to the length of the filaments at break, and *L*_0_ is the original gauge length of the filaments.

### Shear Test of Layer Adhesion

The adhesive bonding of the adjacent layers was tested using the shear test. All scaffolds (*n* = 5) were tested using a homemade shear fixture assembled to a Instron 3344 equipment (Instron, USA) with a 100 N load cell. The tests were applied at a rate of 0.5 mm min^−1^, and the shear stress at failure *τ* was calculated as follows:

(6) $$\tau =\frac{F_{s}}{A_{s}}$$

where *F*_s_ is the applied force at failure, and *A*_s_ refers to the cross‐sectional area of the scaffolds.

### Nanoindentation

Nanoindentation was performed using the NanoTest Vantage (Micro Materials, UK) with a maximum load of 100 mN. The surface hardness and reduced elastic modulus were obtained by measuring at least three different spots on the surface of the filaments.

### Accelerated Degradation

Accelerated degradation tests were performed based on a previously reported protocol.^[^ ^32^, ^77^ ^]^ Briefly, scaffolds (*n* = 5) were cut into approximately 4 mm (*L*) × 4 mm (*W*) × 2.5 mm (*H*) samples. The initial weight was measured in a dry condition, followed by immersing the sample in a 5 m NaOH solution at 37 °C with a degradation time ranging from 4 to120 h. At specific time points, the samples were dried in the fume hood for 48 h and the weight was measured.

### Wettability

The wettability of the scaffolds (*n* = 3) was assessed using a static WCA equipped with a goniometer (KSV Cam 200, Finland). Images were obtained after the ejection of a distilled water droplet onto the top surface of the scaffold at 0 and 30 s and the corresponding contact angles were measured.

### Thermal Analysis: TGA

TGA (Q500, TA Instruments, USA) was performed in a nitrogen atmosphere with a flow rate of 60 mL min^−1^. Scans were performed from room temperature to 560 °C by increasing the temperature at a rate of 10 °C min^−1^. The degradation temperature and the remaining mass of the sample (*n* = 3) were obtained using the Universal Analysis software (TA Instruments, USA).

### DSC

DSC (Q100, TA Instruments, USA) was performed in a nitrogen atmosphere with a flow rate of 50 mL min^−1^. The samples (*n* = 3) were first heated from ‐90 °C to 100 °C at a rate of 10 °C min^−1^, followed by a fast cooling process to ‐90 °C at a rate of 20 °C min^−1^, and eventually submitted to the second heating cycle with the increase of temperature up to 100 °C at a heating rate of 10 °C min^−1^. Melt enthalpy (Δ*H*_m_), melting temperature (*T*_m_), crystallization temperature (*T*_c_), and glass transition temperature (*T*_g_) of PCL were analyzed using the Universal Analysis software (TA Instruments, USA). Crystallinity (*χ*_c_) was calculated according to the following equation:

(7) $$\chi_{c}\left(\%\right)=\frac{\Delta H_{m}}{\Delta H_{m}^{0}}\times \frac{100}{w}$$

where Δ*H*_m_ refers to the melting enthalpy, $\Delta H_{m}^{0}$=139.5 J g^−1^ is the melting enthalpy of PCL with complete crystallization, and *w* is the weight fraction of PCL in the sample.

### XRD

The crystallographic characteristics of PCL in the printed scaffolds were performed through XRD (Ultima IV X‐Ray Diffractometer, Rigaku, Japan) with diffraction angles (*2θ*) ranging from 10° to 50°. The obtained XRD patterns were processed using the OriginPro software (OriginLab Corporation, USA) and the crystallite size (*D*) was calculated by the Scherrer equation:^[^ ^78^ ^]^

(8) $$D=\frac{\kappa \lambda }{\beta \cos\theta }$$

where *κ* = 0.9 is the Scherrer constant; *λ* = 0.154 nm refers to the X‐ray wavelength; *β* is the line broadening at the full width at half maximum (FWHM); and *θ* refers to the Bragg's angle.

### Polarized Microscopy

Polarized microscopy (DM2700M, Leica Microsystems, Germany) was used to evaluate the crystal microstructure of the MPCL and SPCL filaments. The filaments were embedded in an optimal cutting temperature compound prior to freezing and slicing in a cryo‐microtome (CM3050 S, Leica Biosystems, Germany) into 10 µm sections.

### In Vitro Studies: Cell Culture

Scaffolds were sterilized in 80% ethanol for 4 h and rinsed with sterile PBS at least 3 times, and then dried overnight in a sterile tissue culture hood. The hADSCs (Passage 4) were cultured in MesenPRO RS basal cell culture media containing 2% (v/v) growth supplement, 1% (v/v) glutamine, and 1% (v/v) penicillin/streptomycin under standard conditions (37 °C, 5% CO_2_, and 95% humidity). A 0.5 mL of cell suspension containing 25000 cells was added to each scaffold. The cell growth media was changed every 2 d.

For osteogenic differentiation studies, all samples were cultured for 3 d in cell growth media before being replaced with osteogenic differentiation media, assigned as day 1, containing 10% (v/v) osteogenic serum and 1% (v/v) penicillin/streptomycin. The osteogenic assessments were performed at days 3, 7, and 14 and the differentiation media was changed every 2 d.

### Cell Metabolic Activity

The resazurin assay (Alamar Blue) was used to assess the cell seeding efficiency and cell metabolic activity at days 3, 7, and 14. Samples (*n* = 4) were incubated in 0.8 mL of cell culture media, followed by adding 80 µL of 0.01% v/v Alamar blue solution. After 4 h, the absorbance of the sample was read at 570 nm using a microplate reader (Infinite 200, Switzerland) and normalized to the absorbance at 600 nm which was used as a reference wavelength.

### Cell Proliferation

MTT assay was used to assess cell proliferation at days 3, 7, and 14. Scaffolds (*n* = 4) were transferred to a new 24‐well plate followed by adding 150 µL of DMEM and 150 µL of MTT reagent. After incubation for 3 h, 450 µL of MTT solvent was added and shaken in the dark for 15 min. The absorbance was read at 590 nm using a microplate reader (Infinite 200, Switzerland).

### Osteogenic Differentiation

ALP assay was performed according to the manufacturer's instructions at days 3, 7, and 14. Briefly, scaffolds (*n* = 4) were gently washed twice using 1× ALP assay buffer followed by the addition of an appropriate amount of 1× assay buffer mixed with 0.2% v/v Triton X‐100. The samples were subsequently vortexed for 1 min, sonicated for 3 min, and freeze‐thawed twice at ‐80 °C. After the samples were centrifuged at 2500 × *g* for 10 min at 4 °C, the supernatant was collected and mixed with para‐nitrophenyl phosphate substrate solution for 1 h at room temperature. The absorbance was measured at 405 nm using a microplate reader (Infinite 200, Switzerland). The concentration of ALP was obtained from the standard curve and normalized to the total protein concentration determined by BCA.

OCN was measured at days 3, 7, and 14 using a human osteocalcin enzyme‐linked immunosorbent assay (ELISA) kit. Scaffolds (*n* = 4) were washed with PBS twice and lysed in RIPA cell lysis buffer containing a protease inhibitor for 30 min at 4 °C. The lysis solution was centrifuged at 3500 × *g* for 15 min at 4 °C. 25 µL of supernatant was added into a pre‐coated 96‐well plate and mixed with 100 µL of the antibody cocktail for 2 h at room temperature. The samples were then washed three times and mixed with 100 µL of chromogen solution for 30 min in the dark. 100 µL of stop solution was added to each well and the absorbance was measured at 450 nm using a microplate reader (Infinite 200, Switzerland). The OCN concentration was obtained from the standard curve and normalized to the total protein concentration.

### Immunofluorescence Analysis

Confocal immunofluorescence microscopy (SP8, Leica Microsystems, Germany) was used to evaluate cell morphology and mechanotransduction in the scaffolds. The influence of material stiffness on cell behavior and mechanotransduction was semiquantitatively assessed through the expression of paxillin, YAP, and collagen type I in either noninhibited or blebbistatin inhibited scaffolds.

Paxillin, expressed at FAs, and YAP were evaluated by seeding hADSCs onto the scaffolds and cultured for 24 h. The scaffolds were then split into noninhibited and inhibited (treated with 50 × 10^−6^ m of blebbistatin) groups and cultured for a further 24 h. The scaffolds were then fixed in 10% formalin for 25 min and washed in PBS. The scaffolds were permeabilized in 0.1% Triton‐X100 solution for 5 min and blocked in 5% FBS solution for 60 min. The scaffolds were then stained with Alexa Fluor 647 anti‐paxillin antibody with a dilution of 1:200 for 24 h at 4 °C, followed by 1:400 phalloidin for 20 min, and 300 × 10^−9^ m DAPI solution for 5 min. Scaffolds were washed in PBS after each step. Similarly, for YAP staining of the scaffolds a 1:200 dilution anti‐YAP primary antibody was used for 24 h at 4 °C, followed by 1:200 secondary antibody, phalloidin, and DAPI solutions.

For COL‐I staining, the scaffolds were seeded with hADSCs and cultured for 3 d in growth media. The growth media was replaced with osteogenic media (plus 50 × 10^−6^ m blebbistatin in the inhibited group) and cultured for further 3 d. Similar to YAP staining, the scaffolds were then fixed and stained with a 1:200 dilution of anti‐collagen type I primary antibody for 24 h at 4 °C, followed by staining with 1:200 secondary antibody conjugated to Alexa Fluor 647, phalloidin, and DAPI solutions. Images were semiquantitatively analyzed by using the open‐source software CellProfiler following the software guidance.^[^ ^79^, ^80^ ^]^

### Statistical Analysis

Sample size (*n*) was stated in the specific methodology sections. The results were expressed as mean ± SD. Statistical analysis was performed using one‐way analysis of variance (ANOVA) with Tukey multiple comparison test using GraphPad Prism software (Graphpad Software Inc., USA). Differences were considered significant at *P < 0.05, **P < 0.01, ***P < 0.001 and ****P < 0.0001.

## Conflict of Interest

The authors declare no conflict of interest.

## Author Contributions

B.Y.H., Y.X.W., and C.V. contributed equally to this work. Conceptualization: B.Y.H., C.V., and P.B.; Methodology: B.Y.H., Y.X.W., and C.V.; Investigation: B.Y.H., Y.X.W., and C.V.; Visualization: B.Y.H., Y.X.W., and C.V.; Funding acquisition: C.V. and P.Y.B.; Project administration: P.B.; Supervision: P.B.; Writing—original draft: B.Y.H. and Y.X.W.; Writing—review & editing: B.Y.H., Y.X.W., C.V., and P.B.

## Supporting information

Supporting Information

## Data Availability

The data that support the findings of this study are available from the corresponding author upon reasonable request.
