# Supplementary material for: Crystal Growth of 3D Poly(ε‐caprolactone) Based Bone Scaffolds and Its Effects on the Physical Properties and Cellular Interactions
Source: Adv Sci (Weinh). 2022 Nov 17;10(1):2203183. doi: 10.1002/advs.202203183 (PMC9811450; doi:10.1002/advs.202203183)
Supplement: Supplementary file 1 — Supporting Information [file ADVS-10-2203183-s001.pdf]

ADVANCED SCIENCE
Open Access

Supporting Information

for *Adv. Sci.*, DOI 10.1002/advs.202203183

Crystal Growth of 3D Poly(ϵ-caprolactone) Based Bone Scaffolds and Its Effects on the Physical Properties and Cellular Interactions

*Boyang Huang, Yaxin Wang, Cian Vyas and Paulo Bartolo**

Supplementary Information

Crystal growth of 3D poly(ε-caprolactone) based bone scaffolds and its effects on the physical properties and cellular interactions

Boyang Huang, Yaxin Wang, Cian Vyas, Paulo Bartolo *

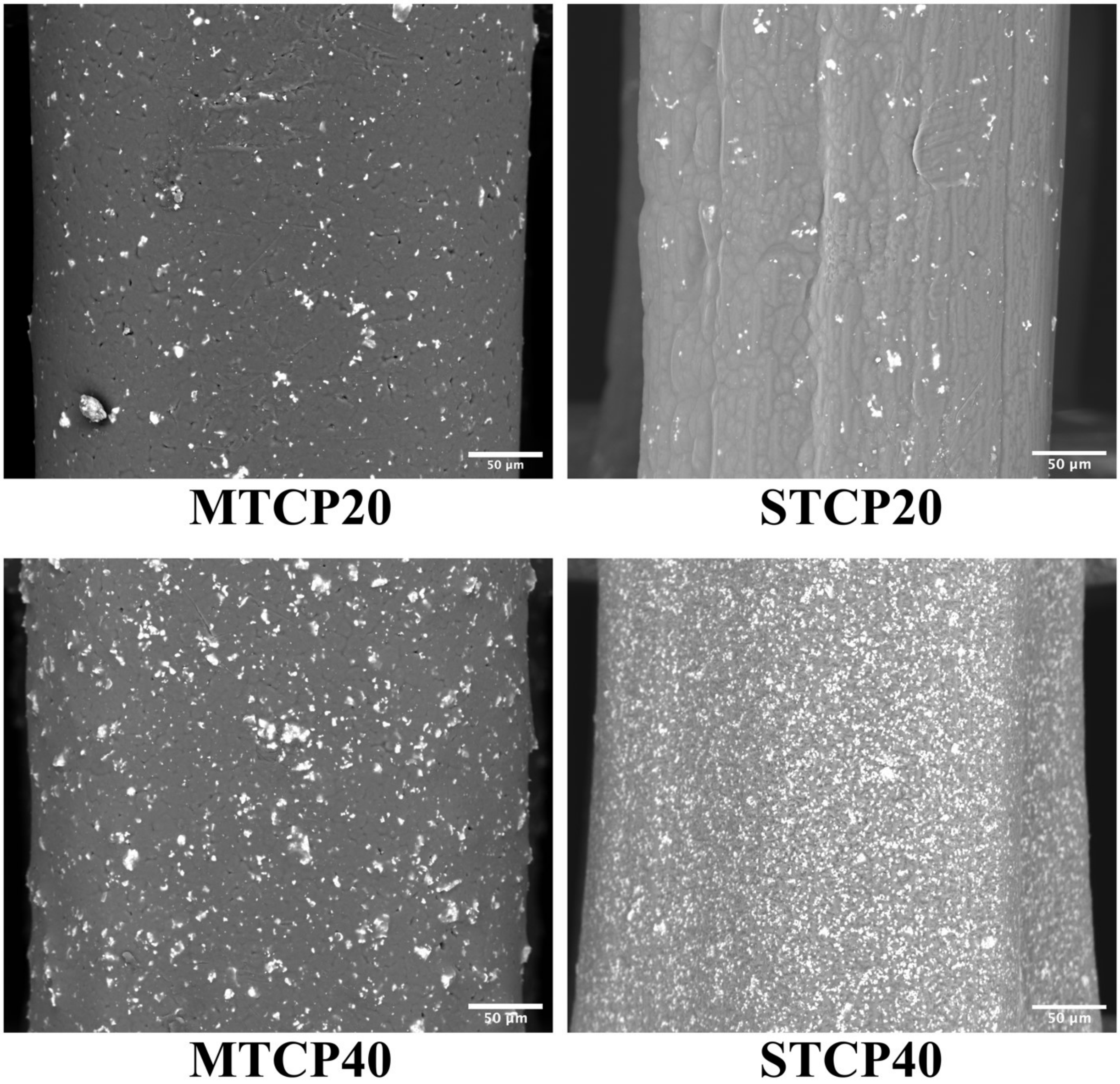

Figure S1. Backscattered electron (BSE) images of MTCP20, STCP20, MTCP40 and STCP40 filaments showing the size and distribution of TCP particles. TCP particle distribution is similar in MTCP20 and STCP20 filaments, however, a more homogenous TCP distribution and smaller particle aggregation are observed in solvent printed filament at 40% wt% loading of TCP. Scale bar: 50 µm.

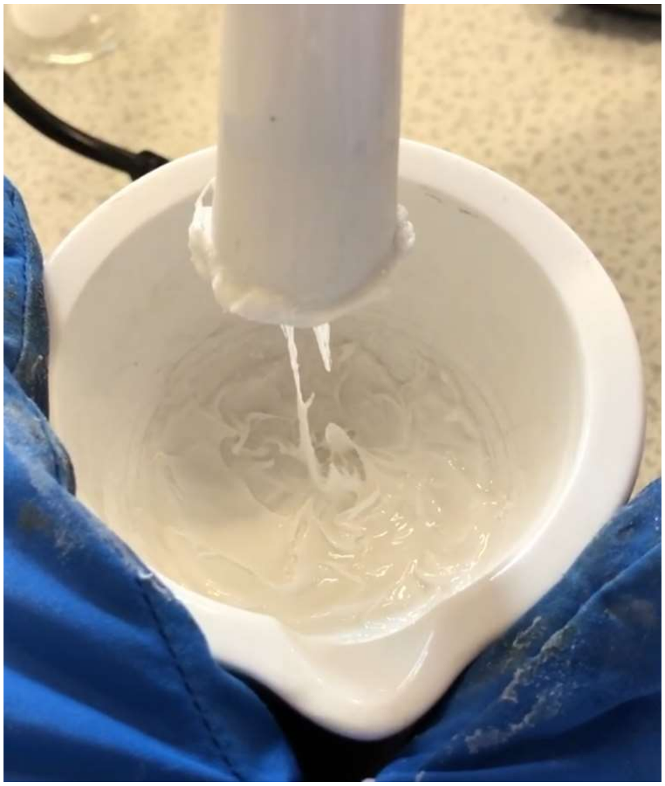

Figure S2. The melt blended composite containing high concentration (50 wt%) of TCP and PCL presenting a poor printability.

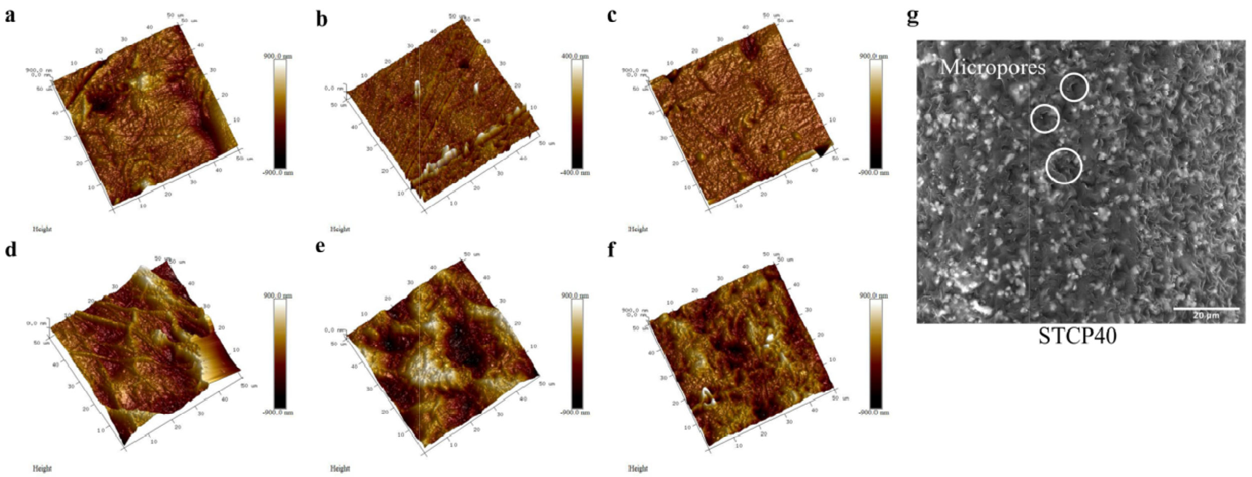

Figure S3. AFM images showing surface topography of **(a)** MPCL, **(b)** MTCP20, **(c)** MTCP40, **(d)** SPCL, **(e)** STCP20 and **(f)** STCP40 (50 μm×50 μm). **(g)** SEM image of STCP40 showing micropores on the STCP40 scaffold (scale bar: 20 μm).

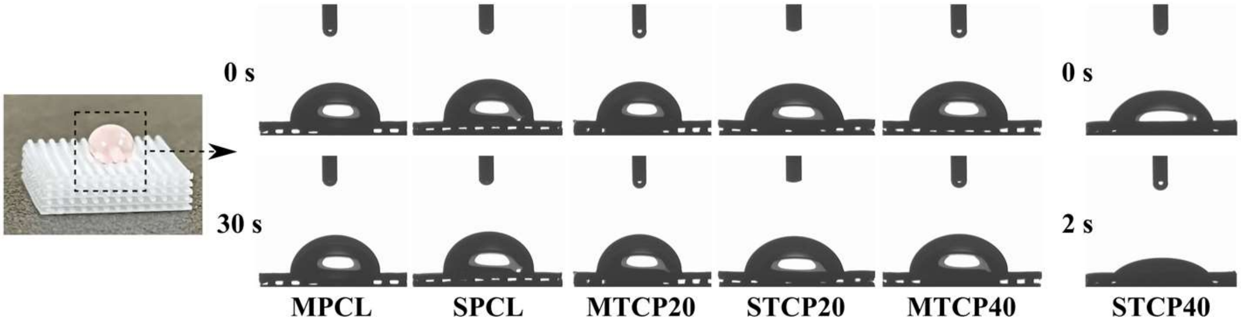

Figure S4. Representative WCA images of scaffolds at 0 s and 30 s, and STCP40 scaffold at 0 s and 2 s showing super hydrophilicity.

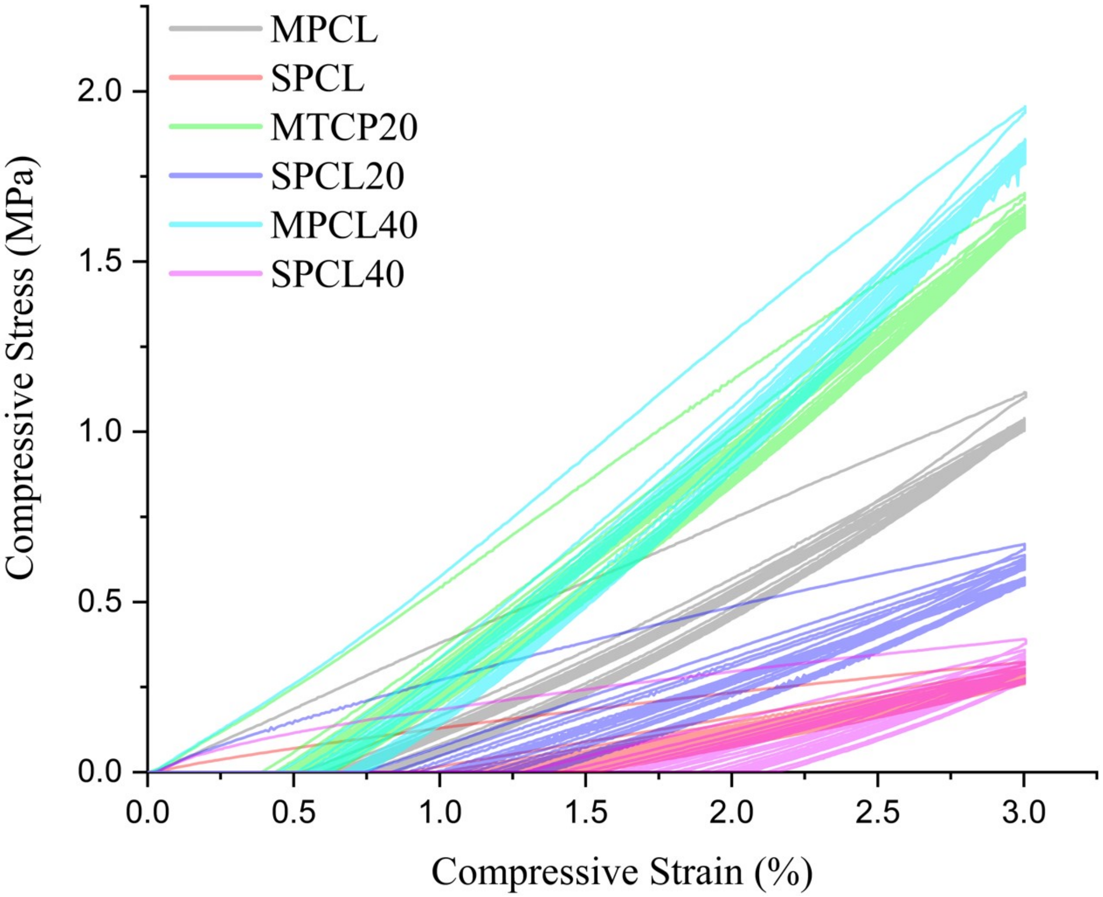

Figure S5. Cyclic compression stress-strain curves for all scaffolds.

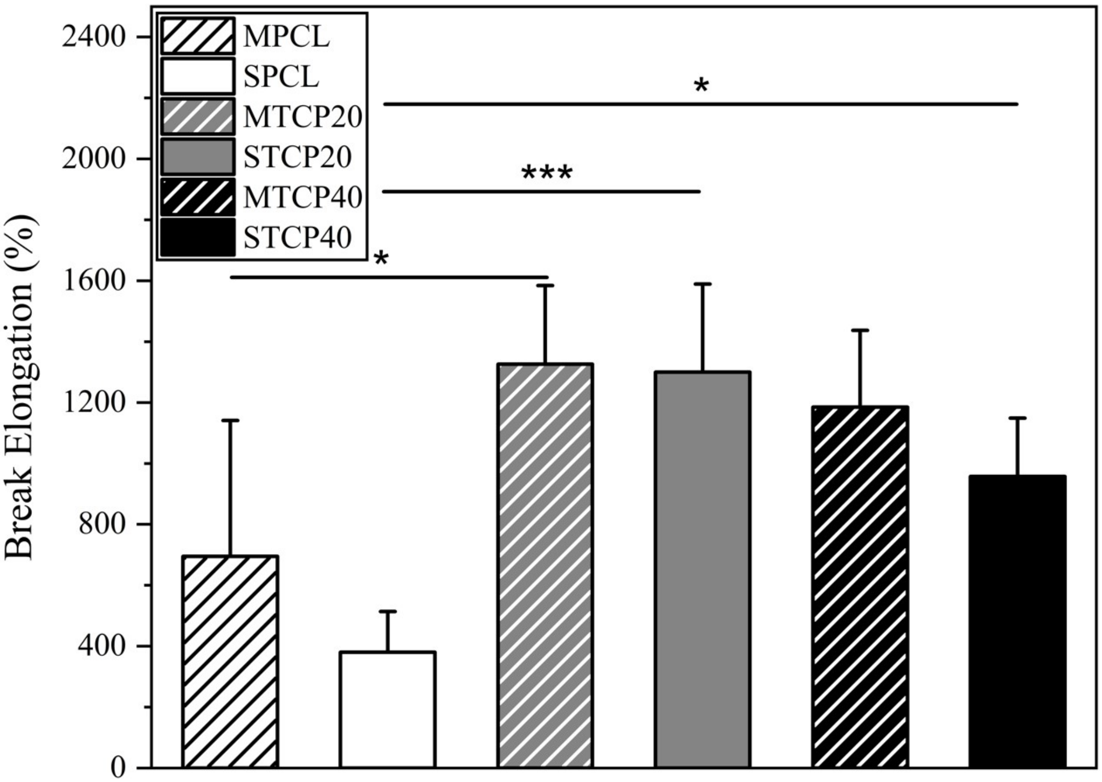

Figure S6. Elongation of melt and solvent printed fibers at break (n=5). Data were presented as mean ± standard deviation. Statistical analysis was performed using one-way analysis of variance (ANOVA). Differences were considered significant at *P < 0.05, **P < 0.01, ***P < 0.001 and ****P < 0.0001.

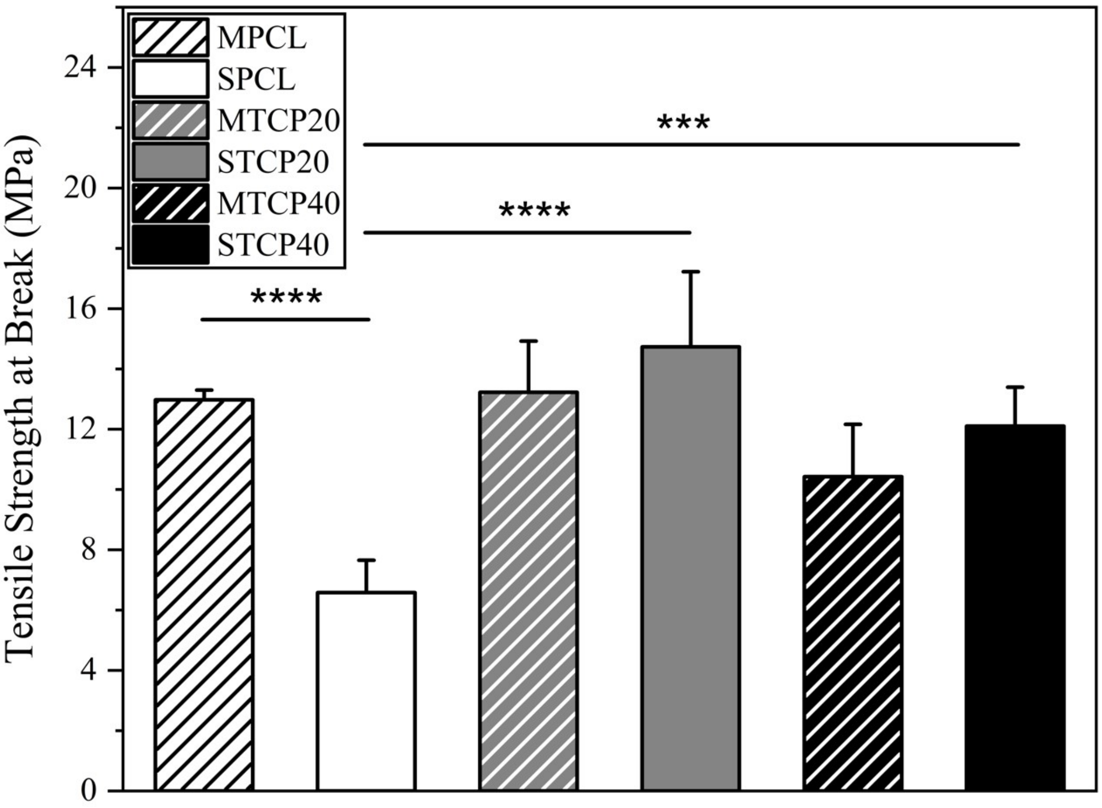

Figure S7. Tensile strength of melt and solvent printed fibers at break (n=5). Data were presented as mean ± standard deviation. Statistical analysis was performed using one-way analysis of variance (ANOVA). Differences were considered significant at *P < 0.05, **P < 0.01, ***P < 0.001 and ****P < 0.0001.

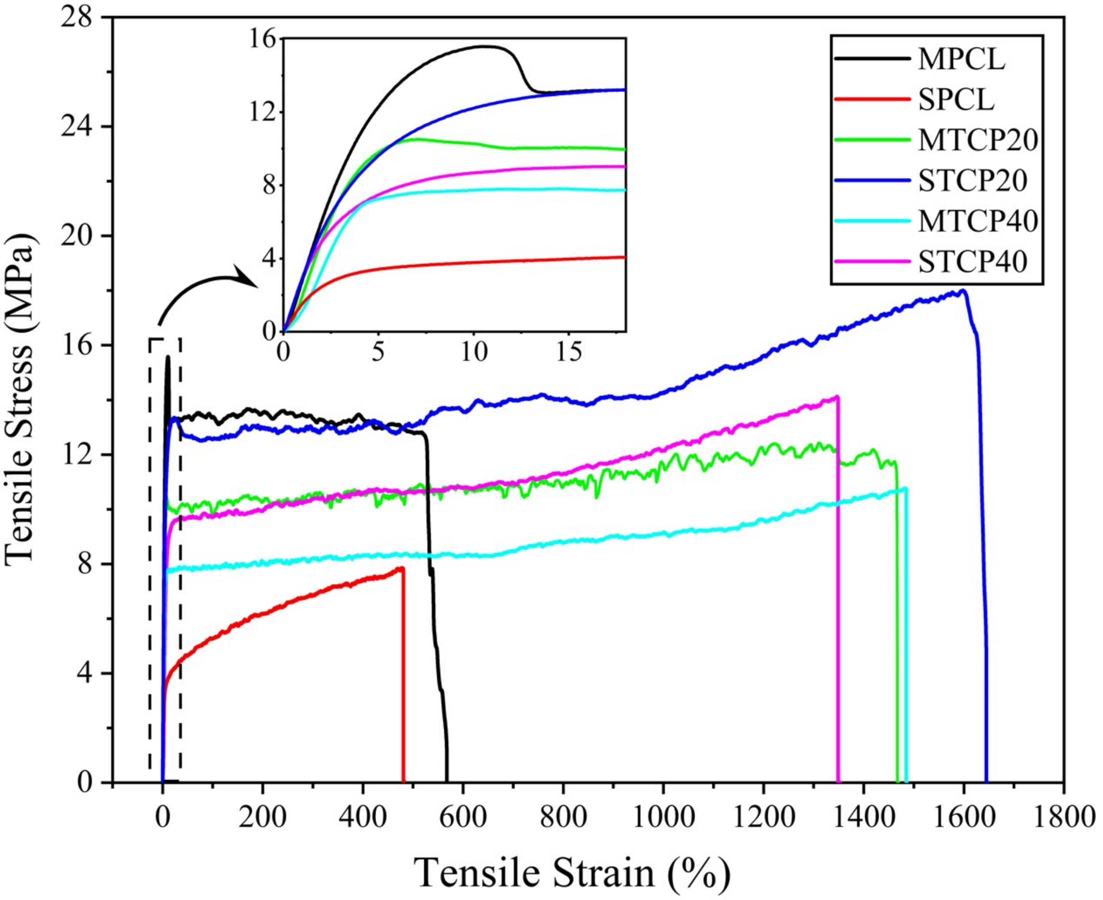

Figure S8. Representative of tensile stress-strain curves all printed fibers.

Table S1. Filament width, pore size, layer thickness and calculated porosity of the 3D printed scaffolds.

	**Filament width (μm)**	**Pore size (μm)**	**Layer thickness (μm)**	**Porosity (%)**
**Designed parameter**	**330**	**350**	**270**	**/**
**MPCL**	309.69±10.24	331.75±12.90	252.07±14.52	53.78±1.37
**SPCL**	322.37±13.01	332.20±29.12	274.29±14.20	53.75±3.18
**MTCP20**	327.43±14.78	331.47±14.33	277.84±10.80	53.88±0.96
**STCP20**	333.19±13.49	328.38±49.17	287.77±20.69	52.31±3.11
**MTCP40**	306.08±12.28	377.46±24.71	201.07±7.98	51.97±1.02
**STCP40**	336.51±7.47	318.34±7.91	276.84±4.90	53.69±3.34

Table S2. TGA results and DSC results obtained from 1st, 2nd heating and cooling curves of melt and solvent printed scaffolds.

	TGA		DSC						
	Measured TCP concentration (wt%)	Degradation temperature (°C)	Melting temperature T_m (°C)		Glass transition temperature T_g (°C)		Crystallization temperature T_c (°C)	Crystallinity X_c (%)	
			1st heating	2nd heating	1st heating	2nd heating	cooling	1st heating	2nd heating
**MPCL**	0.00	372.30±0.98	61.67±1.65	57.47±0.44	-64.77±1.49	-64.46±0.76	20.58±0.66	54.32±1.71	42.34±2.49
**SPCL**	0.00	376.48±0.19	58.02±2.98	54.47±0.30	-66.60±1.90	-68.26±2.43	16.48±2.91	51.23±8.45	41.12±0.60
**MTCP20**	19.93±0.08	371.15±2.18	61.60±0.60	57.63±0.54	-65.56±0.22	-65.55±0.07	24.58±0.91	54.10±1.59	41.42±1.56
**STCP20**	18.63±0.01	374.37±0.57	60.24±0.36	55.16±0.12	-66.66±2.22	-65.71±0.08	17.22±1.91	55.58±8.02	41.39±6.59
**MTCP40**	40.01±0.06	369.90±6.62	62.49±0.79	58.08±1.07	-65.48±0.02	-65.55±0.08	24.63±1.96	53.87±3.49	42.99±3.54
**STCP40**	38.64±0.02	371.12±1.26	58.15±0.09	53.35±0.15	-65.46±0.22	-68.23±4.61	17.67±1.37	56.50±0.47	41.61±0.51
